# Non-additive Effects in Genomic Selection

**DOI:** 10.3389/fgene.2018.00078

**Published:** 2018-03-06

**Authors:** Luis Varona, Andres Legarra, Miguel A. Toro, Zulma G. Vitezica

**Affiliations:** ^1^Departamento de Anatomía, Embriología y Genética Animal, Universidad de Zaragoza, Zaragoza, Spain; ^2^Instituto Agroalimentario de Aragón (IA2), Zaragoza, Spain; ^3^Génétique Physiologie et Systèmes d'Elevage (GenPhySE), Institut National de la Recherche Agronomique de Toulouse, Castanet-Tolosan, France; ^4^Departamento Producción Agraria, ETS Ingeniería Agronómica, Alimentaria y de Biosistemas, Universidad Politécnica de Madrid, Madrid, Spain; ^5^Génétique Physiologie et Systèmes d'Elevage (GenPhySE), Université de Toulouse, Castanet-Tolosan, France

**Keywords:** genomic selection, dominance, epistasis, crossbreeding, genetic evaluation

## Abstract

In the last decade, genomic selection has become a standard in the genetic evaluation of livestock populations. However, most procedures for the implementation of genomic selection only consider the additive effects associated with SNP (Single Nucleotide Polymorphism) markers used to calculate the prediction of the breeding values of candidates for selection. Nevertheless, the availability of estimates of non-additive effects is of interest because: (i) they contribute to an increase in the accuracy of the prediction of breeding values and the genetic response; (ii) they allow the definition of mate allocation procedures between candidates for selection; and (iii) they can be used to enhance non-additive genetic variation through the definition of appropriate crossbreeding or purebred breeding schemes. This study presents a review of methods for the incorporation of non-additive genetic effects into genomic selection procedures and their potential applications in the prediction of future performance, mate allocation, crossbreeding, and purebred selection. The work concludes with a brief outline of some ideas for future lines of that may help the standard inclusion of non-additive effects in genomic selection.

## Introduction

Through his experiments on pea plants, Gregor Mendel (1866) realized that some traits are dominant over others (for example “round peas” were dominant over “wrinkled peas”). In Mendel's own words: “As a rule, hybrids do not represent the form exactly intermediate between the parental strains… Those traits that pass into hybrid association entirely or almost entirely unchanged, thus themselves representing the traits of the hybrid, are termed “dominating,” and those that become latent in the association, “recessive””. Shortly after the rediscovery of Mendel's rules, it was observed that, in some cases, the addition of the individual action of genes could not explain the mode of inheritance, and Bateson (1909) coined the term “epistasis” to describe the cases in which the actions of two or more genes interact. A distinction must be drawn between biological (functional) genetic effects that correspond to the Mendelian definition (i.e., dominance means that the heterozygote value is higher or lower than the mean of homozygous genotypes) and statistical (population or weighted) effects which depend on allelic frequencies. In the latter, the relevant issue is the contribution of non-additive effects to genetic variance. Some authors argue that non-additive genetic effects may be a general phenomenon whose understanding is important for gaining more knowledge on the nature of quantitative traits, but whose contribution to variance is negligible (Crow, 2010).

From the perspective of quantitative genetics, Fisher (1918) conceived the infinitesimal model which postulates that a very large number of unlinked genes control the genetic variation of quantitative traits. He described the resemblance between relatives in a pure additive model which was quickly extended to incorporate dominance (Fisher, 1918; Wright, 1921). Resemblance between relatives including epistatic effects of second and higher order was also described (Cockerham, 1954; Kempthorne, 1954). However, whilst the formulation of the infinitesimal model in the additive context is evident, its interpretation is not clear when non-additive effects are included (Barton et al., 2017).

The main goal of animal or plant breeding is to identify, select and mate the best individuals of a breeding stock in order to maximize performance in future generations (Falconer and McKay, 1996; Bernardo, 2010). The procedure for computing the breeding values (genetic evaluation) of candidates for selection plays a crucial role. Traditionally, these methods use phenotypic and genealogical information, such as the selection index (Hazel, 1943) or the Best Linear Unbiased Predictor (Henderson, 1973) and rely on the foundations of the infinitesimal model (Fisher, 1918).

Nevertheless, non-additive genetic effects have been ignored in the genetic evaluation of livestock for several reasons: (i) the lack of informative pedigrees, such as large full-sib families; (ii) the calculations involved are more complex; (iii) the fact that statistical additive variance captures biological dominance or higher order interaction effects (Hill, 2010); and, (iv) the difficulty in using dominant values in practice (mate allocation). As a consequence, estimates of non-additive genetic variances are scarce in livestock populations (Misztal et al., 1998; Nguyen and Nagyné-Kiszlinger, 2016).

## Genomic selection

Since the late 80s and 90s, developments in molecular genetics resulted in a set of neutral molecular markers, such as microsatellites, that were commonly used to detect QTL (Quantitative Trait Loci) in almost all livestock populations. The objective of those studies was to identify polymorphic markers or genes associated with phenotypic variation of traits of interest (www.animalgenome.org/QTL), with the ultimate goal of using them in Marker or Gene Assisted Selection (Dekkers, 2004). However, these strategies became obsolete with the advent of dense genotyping devices (Gunderson et al., 2005) that provided a very large amount of SNP (Single Nucleotide Polymorphism) and that allowed the development of genomic selection (GS) models (Meuwissen et al., 2001).

Genomic selection has become a very successful strategy for the prediction of the breeding values of candidates for selection and has revolutionized the field of animal breeding over the past decade. The basic idea of GS is to develop the following linear model:

$$y_{i}=\mu +\sum_{j\;=\;1}^{n}t_{ij}a_{j}+e_{i}$$

The model explains the phenotypic data of *m* individuals (*y*_*i*_) with *i* = 1 …*m* (or transformations of data, such as daughter yield deviations) by the effects associated with a very large number (*n*) of SNP (*a*_*j*_) with *j* = 1 …*n*. Moreover, *t*_*ij*_ is the genotypic configuration (coded additively, e.g., Falconer and McKay, 1996) of the *ith* individual and for the *jth* SNP (0, 1, and 2 for A_1_A_1_, A_1_A_2_, and A_2_A_2_ genotypes, respectively), and *e*_*i*_ is the residual. Furthermore, the prediction of individual breeding values ($\hat{u_{i}}$) of the candidates for selection can be calculated *a posteriori* from marker effect estimates as $\hat{u_{i}}=\overset{n}{\underset{j\;=\;1}{\sum }}t_{ij}\hat{a_{j}}$.

A significant limitation for implementation is that most genomic evaluation models suffer the statistical problem of a larger number of parameters (*n*) that must be estimated from a smaller number of data (*m*). The most common method employed for resolving this problem is the use of some type of regularization of SNP marker effects (Gianola, 2013). Several approaches have been suggested, ranging from a simple Gaussian regularization (Meuwissen et al., 2001) to more complex models that involve *t* shaped (Meuwissen et al., 2001), double exponential (De los Campos et al., 2009b), mixtures of distributions (Meuwissen et al., 2001; Habier et al., 2011; Erbe et al., 2012), or non-parametric or semi-parametric approaches (Gonzalez-Recio et al., 2014). The predictive ability of all these approaches depends on the genetic architecture of the traits being analyzed (Daetwyler et al., 2010), although for polygenic traits, all approaches offer similar results (Wang et al., 2015).

An interesting property of the assumption of a Gaussian prior distribution for marker effects (Random Regression BLUP—RR-BLUP) is that the GS model can be reformulated in terms of individual (animal) effects, using the equations of the Henderson's classic Mixed Model that provide breeding values for all individuals, including candidates for selection (Genomic BLUP or GBLUP). The only difference with standard mixed model equations is that the numerator relationship matrix (**A**) is replaced by the genomic relationship matrix (**G**), as defined by VanRaden (2008). In addition, this approach can be extended for the genetic evaluation of non-genotyped individuals in the Single-Step approach (Aguilar et al., 2010), facilitating the integration of GS procedures in the genetic evaluation of candidates for selection in most livestock breeding programmes. More recently, Fernando et al. (2014) described a Bayesian procedure that can also simultaneously evaluate genotyped and non-genotyped individuals and allows the use of alternative regularization procedures. Nevertheless, computational costs are markedly higher with the Bayesian model than with the Single-Step approach.

Despite the regularization procedure, the genomic evaluation methods are based on the evaluation of marker substitution effects through the construction of the covariates (*t*_*ij*_*)* or the **G** matrix (above). The additive (or breeding) values capture a large part of dominant and higher-order interaction effects (Hill et al., 2008; Crow, 2010; Hill, 2010). Substitution effects that capture dominance and epistatic functional effects are not necessarily stable across generations or populations due to changes in allelic frequencies. In any case, only additive values (substitution effects) contribute to breeding values and are therefore expressed in the next generation. However, estimates of non-additive genetic effects may be of relevance because: (i) they may contribute to increasing the accuracy of prediction of breeding values and the response to selection (Toro and Varona, 2010; Aliloo et al., 2016; Duenk et al., 2017); (ii) they allow the definition of mate allocation procedures between candidates for selection (Maki-Tanila, 2007; Toro and Varona, 2010; Aliloo et al., 2017); and (iii) they can be used to benefit from non-additive genetic variation through the definition of appropriate crossbreeding or purebred breeding schemes (Maki-Tanila, 2007; Zeng et al., 2013).

## Genomic selection models with dominance

The simplest approach for the inclusion of dominance in genomic selection models is to extend the basic model with the inclusion of a dominance effect (Toro and Varona, 2010; Su et al., 2012) associated to each SNP marker:

$$y_{i}=\mu +\sum_{j\;=\;1}^{n}t_{ij}a_{j}+\sum_{j\;=\;1}^{n}c_{ij}d_{j}+e_{i}$$

where *y*_i_ is the phenotypic value of the *ith* individual and μ is the population mean. For each of the *n* SNP markers, *a*_*j*_ and *d*_*j*_ are the additive and dominance effects for the *jth* marker, respectively. The covariates *t*_*ij*_ and *c*_*ij*_ are 2, 1, and 0 (coded additively) and 0, 1, and 0 (coded in a “biological dominant” manner) for the genotypes A_1_A_1_, A_1_A_2_, A_2_A_2_ of each marker, respectively. In some ways, pedigree-based models for dominance were based on “expected” dominant relationships. Thus, genomic models are based on “observed” heterozygotes. However, when using this model it should be noted that that *a*_*j*_ is no longer the marker substitution effect, but the “biological” additive genotypic effect and individual breeding values are not predicted. In fact, the partition of variance in statistical components due to additivity, dominance, and epistasis does not reflect the “biological” (or “functional”) effect of the genes although it is useful for prediction and selection (Huang and Mackay, 2016). The model was reformulated in terms of breeding values and dominance deviations (Falconer and Mackay, 1996) by Vitezica et al. (2013) after the assumption of a Hardy-Weinberg equilibrium within each:

$$y_{i}=\mu +\sum_{j\;=\;1}^{n}w_{ij}\alpha_{j}+\sum_{j\;=\;1}^{n}g_{ij}d_{j}+e_{i}$$

where

$$w_{ij}=\{\begin{array}{c} \left(2-2p_{j}\right)\\ \left(1-2p_{j}\right)\\ -2p_{j} \end{array}\;\begin{array}{c} A_{1}A_{1}\\ A_{1}A_{2}\\ A_{2}A_{2} \end{array}\;g_{ij}=\{\begin{array}{c} -2q_{j}^{2}\\ 2p_{j}q_{j}\\ -2p_{j}^{2} \end{array}\;\begin{array}{c} A_{1}A_{1}\\ A_{1}A_{2}\\ A_{2}A_{2} \end{array}$$

and α_*j*_ = *a*_*j*_ + *d*_*j*_ (*q*_*j*_ − *p*_*j*_) is now the allelic substitution effect and *p*_*j*_ and *q*_*j*_ are the allelic frequencies for A_1_ and A_2_ for the *jth* SNP marker. The genetic variance due to a single locus is:

$$\sigma_{Gj}^{2}=2p_{j}q_{j}{\left[a_{j}+d_{j}\left(q_{j}-p_{j}\right)\right]}^{2}+{\left(2p_{j}q_{j}d_{j}\right)}^{2}$$

where the additive variance is $\sigma_{Aj}^{2}=2p_{j}q_{j}{\left[a_{j}+d_{j}\left(q_{j}-p_{j}\right)\right]}^{2}=2p_{j}q_{j}\alpha_{j}^{2}$ and the dominance variance is $\sigma_{Dj}^{2}={\left(2p_{j}q_{j}d_{j}\right)}^{2}$ and the multilocus variances, under linkage equilibrium (LE), are $\sigma_{G}^{2}=\overset{n}{\underset{j\;=\;1}{\sum }}\sigma_{Gj}^{2}$, $\sigma_{A}^{2}=\overset{n}{\underset{j\;=\;1}{\sum }}\sigma_{Aj}^{2}\;\text{and}\;\sigma_{D}^{2}=\overset{n}{\underset{j\;=\;1}{\sum }}\sigma_{Dj}^{2}$. In fact, “biological” (in terms of genotypic additive and dominant values) and “statistical” (in terms of breeding values and dominance deviations) models are equivalent parameterisations of the same model (Vitezica et al., 2013), and the following expressions:

$$\begin{array}{l} \sigma_{A}^{2}=\sum_{j\;=\;1}^{n}\left(2p_{j}q_{j}\right)\sigma_{a}^{2}+\sum_{j\;=\;1}^{n}\left(2p_{j}q_{j}{\left(q_{j}-p_{j}\right)}^{2}\right)\sigma_{d}^{2}\\ \sigma_{A}^{2}=\sum_{j\;=\;1}^{n}\left(2p_{j}q_{j}\right)\sigma_{a}^{2}\\ \sigma_{D}^{2}=\sum_{j\;=\;1}^{n}\left(4p_{j}^{2}q_{j}^{2}\right)\sigma_{d}^{2}\\ \sigma_{D}^{2}=\sum_{j\;=\;1}^{n}\left(2p_{j}q_{j}\left(1-2p_{j}q_{j}\right)\right)\sigma_{d}^{2} \end{array}$$

that can be used to switch variance components estimates between “biological” ($\sigma_{A^{*}}^{2}$ and $\sigma_{D^{*}}^{2}$) and “statistical” ($\sigma_{A}^{2}$ and $\sigma_{D}^{2}$) models. It can be verified that $\sigma_{A}^{2}+\sigma_{D}^{2}=\sigma_{A^{*}}^{2}{+\;\sigma }_{D^{*}}^{2}$. In addition, if *p* = *q* = 0.5, all variances are identical and if *d* = 0, $\sigma_{A}^{2}\;=\;\sigma_{A^{*}}^{2}$. A further generalization can be also achieved to avoid the requirements of the Hardy-Weinberg equilibrium (Vitezica et al., 2017), by following the NOIA model (Alvarez-Castro and Carlborg, 2007) by replacing *w*_*ij*_ and *g*_*ij*_ with:

$$\begin{array}{l} w_{ij}=\{\begin{array}{c} -\left(-p_{12j}-2p_{22j}\right)\\ -\left(1-p_{12j}-2p_{22j}\right)\\ -\left(2-p_{12j}-2p_{22j}\right) \end{array}\;\begin{array}{c} A_{1}A_{1}\\ A_{1}A_{2}\\ A_{2}A_{2} \end{array}\\ g_{ij}=\{\begin{array}{c} -\frac{2p_{12j}p_{22j}}{p_{11j}\;+\;p{\;}_{22j}-{\left(p_{11j}-p_{22j}\right)}^{2}}\;A_{1}A_{1}\\ \frac{4p_{11j}p_{22j}}{p_{11j}\;+\;p{\;}_{22j}\;-\;{\left(p_{11j}\;-\;p_{22j}\right)}^{2}}\;A_{1}A_{2}\\ -\frac{2p_{11j}p_{12j}}{p_{11j}\;+\;p{\;}_{22j}-{\left(p_{11j}-p_{22j}\right)}^{2}}\;A_{2}A_{2} \end{array} \end{array}$$

where, *p*_11*j*_, *p*_12*j*_, and *p*_22*j*_ are the genotypic frequencies for *A*_1_*A*_1_, *A*_1_*A*_2_, and *A*_2_*A*_2_ at the *jth* SNP marker, respectively.

Note that all these models require a regularization process for additive and dominance effects. The simplest approach is to expand the RR-BLUP by the assumption of a prior Gaussian distribution for the additive and dominance effects. It is feasible to assume any other kind of prior distribution for the dominance (as described above) and the additive effects (Acevedo et al., 2015). However, a major advantage of using a Gaussian prior distribution is that the model can be easily transformed into Henderson's Mixed Model equations by using the definition of additive (**G**) and dominance covariance matrices (**D**), as suggested by Vitezica et al. (2013).

Genomic selection models with dominance have been tested in several populations, including dairy cattle (Ertl et al., 2014; Aliloo et al., 2016; Jiang et al., 2017), pigs (Esfandyari et al., 2016; Xiang et al., 2016), sheep (Moghaddar and van der Werf, 2017), and layers (Heidaritabar et al., 2016) with ambiguous results. Jiang et al. (2017) found a negligible percentage of variation explained by dominance effects for productive life in a Holstein cattle population, although Ertl et al. (2014) suggested that dominance may suppose up to 39% of the total genetic variation for Somatic Cell Score in a population of Fleckvieh cattle. In general, the increase in the accuracy of additive breeding values by including dominance was scarce, with the exception of Aliloo et al. (2016).

## Dominance and inbreeding depression (or heterosis)

The classical theory of quantitative genetics (Falconer and Mackay, 1996) postulates that inbreeding depression (or heterosis) occurs due to directional dominance. However, the presence of directional dominance (i.e., a higher percentage of positive than negative dominant effects) is in sharp contrast to the assumptions of the procedures described above that use symmetric prior distributions. This drawback can be overcome by the assumption of a mean of dominant effects that is different from zero, e.g., *E*(*d*) = μ_*d*_, as proposed by Xiang et al. (2016). The standard model can be reformulated as:

$$\begin{array}{l} y_{i}=\mu +\sum_{j\;=\;1}^{n}t_{ij}a_{j}\;+\;\sum_{j\;=\;1}^{n}c_{ij}\left[d_{j}^{*}\;+\;\mu_{d}\right]\;+\;e_{i}\\ \;=\;\mu +\sum_{j\;=\;1}^{n}t_{ij}a_{j}\;+\;\sum_{j\;=\;1}^{n}c_{ij}d_{j}^{*}\;+\;\;\sum_{j\;=\;1}^{n}c_{ij}\mu_{d}+\;\;e_{i} \end{array}$$

where $d_{j}^{*}=d_{j}-\mu_{d}$, then *E*(d^*^) = 0. It should be noted the term $\overset{n}{\underset{j\;=\;1}{\sum }}c_{ij}\mu_{d}$ is an average of dominance effects for the *ith* individual, because *c*_*ij*_ has a value of 1 for heterozygous loci and 0 for homozygous. Inbreeding (or full homozygosity) coefficients *f*_*i*_ can be calculated as:

$$f_{i}=1-\;\frac{\sum_{j\;=\;1}^{n}c_{ij}}{n}$$

So, $\overset{n}{\underset{j\;=\;1}{\sum }}c_{ij}\mu_{d}=\left(1-f_{i}\right)n\mu_{d}=n\mu_{d}-f_{i}n\mu_{d}$. The first term *nμ*_*d*_ is absorbed in the overall mean of the model (μ), and the second (−*f*_*i*_*nμ*_*d*_) corresponds to a covariate *b* = −*nμ*_*d*_ associated with inbreeding (*f*_*i*_). This covariate can be seen as inbreeding depression (if it has a detrimental effect) caused by genomic inbreeding. In addition, it can be also implemented in the GBLUP models described above with the introduction of a covariate within the mixed model equations.

Nonetheless, it assumes that the expected mean of the dominance effects is the same for all markers. In the literature, there are signs that the decrease in performance is associated heterogeneously within the genomic regions (Pryce et al., 2014; Howard et al., 2015; Saura et al., 2015). Models that consider alternative means of dominance effects within genomic regions may be useful to model inbreeding depression in a more appropriate way.

An alternative approach to explain the phenomenon of inbreeding depression (or heterosis) is the consideration of a possible relationship between additive and dominance biological effects (Wellmann and Bennewitz, 2011). There is theoretical proofs (Caballero and Keightley, 1994) and empirical evidence (Bennewitz and Meuwissen, 2010) that supports this argument. Wellmann and Bennewitz (2012) expanded the “biological” model described above with regularization procedures that allows for this dependence. They defined up to four models (Bayes D0 to D3) based on the Bayes C approach (Verbyla et al., 2009). The last two models (Bayes D2 and D3) included dependencies between genotypic additive and dominance effects. In the first (D2), the dependence was modeled through the prior variance of the dominance effects (*Var* (*d*||*a*|)) and in the second (D3), they further expanded it to the prior mean (*E* (*d*||*a*|)), where |*a*| is the absolute value of the additive effect. Implementation of these models is extremely complex and they have not been thoroughly tested (Bennewitz et al., 2017).

## Imprinting

Another source of non-additive genetic variation is genomic imprinting (Reik and Walter, 2001). This involves total or partial inactivation of paternal and maternal alleles. Following the quantitative model established by Spencer (2002), Nishio and Satoh (2015) put forward two alternative genomic selection models to include imprinting effects. The first extends the “statistical” model with dominance (in terms of breeding values and dominance deviations) as:

$$y_{i}=\mu +\sum_{j\;=\;1}^{n}w_{ij}\alpha_{j}+\sum_{j\;=\;1}^{n}g_{ij}d_{j}+\sum_{j\;=\;1}^{n}r_{ij}i_{j}+e_{i}$$

where

$$\begin{array}{l} w_{ij}=\{\begin{array}{c} 2-2p_{j}\\ 1-2p_{j}\\ 1-2p_{j}\\ -2p_{j} \end{array}\;\begin{array}{c} A_{1}A_{1}\\ A_{1}A_{2}\\ A_{2}A_{1}\\ A_{2}A_{2} \end{array}\\ g_{ij}=\{\begin{array}{c} -2q_{j}^{2}\\ 2p_{j}q_{j}\\ 2p_{j}q_{j}\\ -2p_{j}^{2} \end{array}\;\begin{array}{c} A_{1}A_{1}\\ A_{1}A_{2}\\ A_{2}A_{1}\\ A_{2}A_{2} \end{array}\text{ and }r_{ij}=\{\begin{array}{c} 0\\ 1\\ -1\\ 0 \end{array}\;\begin{array}{c} A_{1}A_{1}\\ A_{1}A_{2}\\ A_{2}A_{1}\\ A_{2}A_{2} \end{array} \end{array}$$

and *i*_*j*_ is the imprinting effects associated with *jth* marker. The second alternative proposed the distribution of the genetic effects into paternal (*p*_*j*_) and maternal (*m*_*j*_) gametic effects and a dominance deviation.

$$y_{i}=\mu +\sum_{j\;=\;1}^{n}l_{ij}p_{j}+\sum_{j\;=\;1}^{n}j_{ij}m_{j}+\sum_{j\;=\;1}^{n}g_{ij}d_{j}+e_{i}$$

where

$$l_{ij}=j_{ij}=\{\begin{array}{c} q_{j}\\ -\left(1-q_{j}\right) \end{array}\;\begin{array}{c} A_{1}\\ A_{2} \end{array}$$

These models have been implemented in some studies with livestock data: (Hu et al., 2016) did not find an increase in predictive ability when imprinting effects were included in the model. In addition, estimates of the percentage of phenotypic variation caused by imprinting were small and ranged between 1.3 and 1.4% in pigs (Guo et al., 2016) and from 0.2 to 2.1% in dairy cattle (Jiang et al., 2017). However, this latter study reported that imprinting effects supposed more than 20% of the total genetic variance in some reproductive traits, like pregnancy or conception rate.

## Epistasis

The last and most complex source of non-additive genetic variation is the epistatic interactions between two or more genes. An immediate approach for genomic evaluation including epistatic interactions is to define an explicit model by including pairwise or higher order epistatic effects:

$$\begin{array}{l} y_{i}=\mu +\sum_{j\;=\;1}^{n}t_{ij}a_{j}+\sum_{j\;=\;1}^{n}c_{ij}d_{j}+\sum_{j\;=\;1}^{n}\sum_{k\;=\;1}^{n}t_{ij}t_{ik}aa_{jk}\\ \;+\sum_{j\;=\;1}^{n}\sum_{k\;=\;1}^{n}t_{ij}g_{ik}ad_{jk}+\sum_{j\;=\;1}^{n}\sum_{k\;=\;1}^{n}g_{ij}g_{ik}dd_{jk}\\ \;+\sum_{j\;=\;1}^{n}\sum_{k\;=\;1}^{n}\sum_{l\;=\;1}^{n}t_{ij}t_{ik}t_{il}aaa_{jkl}+\sum_{j\;=\;1}^{n}\sum_{k\;=\;1}^{n}\sum_{l\;=\;1}^{n}t_{ij}t_{ik}g_{il}aad_{jkl}\\ \;+\sum_{j\;=\;1}^{n}\sum_{k\;=\;1}^{n}\sum_{l\;=\;1}^{n}t_{ij}g_{ik}g_{il}add_{jkl}+\sum_{j\;=\;1}^{n}\sum_{k\;=\;1}^{n}\sum_{l\;=\;1}^{n}g_{ij}g_{ik}g_{il}ddd_{jkl}\\ \;+\ldots +e_{i} \end{array}$$

where *aa*_*jk*_, *ad*_*jk*_, and *dd*_*jk*_ are second order additive x additive, additive x dominant and dominant x dominant epistatic effects between the *jth* and *kth* SNP effects and *aaa*_*jkl*_, *aad*_*jkl*_, *add*_*jkl*_ and *ddd*_*jk*_ are third order additive x additive x additive, additive x additive x dominant, additive x dominant x dominant and dominant x dominant x dominant epistatic effects. Despite the method of regularization used, the number of parameters to estimate is extremely large. Consequently, the computational requirements are enormous and the amount of information available, in the statistical sense, for the estimation of each epistatic effect is very small. Therefore, the most efficient (at least from a computational point of view) method for including epistatic interactions in genomic selection models is to define appropriate covariance matrices between individual effects, in the same way that the standard GBLUP model uses the genomic relationship matrix, but, in this case, taking into account the interactive nature of the genetic effects. There are two main approaches in the published literature: (1) the definition of genomic relationship matrices that consider epistatic interactions (Varona et al., 2014; Martini et al., 2016; Vitezica et al., 2017), and (2) the application of Kernel-based statistical methods (Gianola et al., 2006; de los Campos et al., 2009a; Morota and Gianola, 2014).

This simplest method for defining genomic relationship matrices is the *extended GBLUP model (EGBLUP)*, described by Jiang and Reif (2015) and Martini et al. (2016). These authors start from a reduced version of the “biological” model:

$$y_{i}=\mu +\sum_{j\;=\;1}^{n}t_{ij}a_{j}+\sum_{j\;=\;1}^{n}\sum_{k\;=\;1}^{n}t_{ij}t_{ik}aa_{jk}+e_{i}$$

and they define an equivalent model:

$$y=1\mu +g_{1}+g_{2}+e$$

where μ is the general mean, **y** is the vector of phenotypic data and **e** is the vector of the residuals. In addition, the model includes one “biologically” additive (**g**_1_) and one epistatic (**g**_2_) multivariate Gaussian term with the following distributions:

$$g_{1}\sim N\;\left(0,G_{1}\sigma_{g1}^{2}\right)\;g_{2}\sim N\;\left(0,G_{2}\sigma_{g2}^{2}\right)$$

Where **G_1_ = TT′** and **G_2_ = G_1_**° **G_1_** being:

$$T=\left[\begin{array}{c} t_{11}\cdots t_{1n}\\ \;\vdots \;\ddots \;\vdots \\ t_{k1}\cdots t_{kn} \end{array}\right]$$

and the Hadamard product. Moreover, *n* is the number of SNP markers and *k* the number of individuals. However, with this model the additive and epistatic effects are not orthogonal and dominant effects are not included. Therefore, it can only be used for prediction of the phenotypes and not for the estimation of variance components (Martini et al., 2016). To avoid this inconvenience, Varona et al. (2014) and Vitezica et al. (2017) developed a full orthogonal model. They start with the expansion of the individual genotypic effect into additive, dominance and epistatic effects:

$$\begin{array}{l} y=1\mu +g+e=1\mu +g_{A}+g_{D}+\;\sum_{i\;=\;A,D}\sum_{j\;=\;A,D}g_{ij}\\ \;+\;\sum_{\;i\;=\;A,D}\sum_{j\;=\;A,D}\sum_{k\;=\;A,D}g_{ijk}\;+\ldots +\;e \end{array}$$

Where **g** is the vector of the individual genotypic effects, **g_A_** is the vector of additive effects, **g_D_** the vector of individual dominance effects, **g_ij_** is the second order epistatic effects, **g_ijk_** the third order epistatic effects and so on. For simplicity, each individual effect is defined by the sum of SNP (or combination of SNP) effects **h** with equal prior Gaussian variability and weighted by an incidence matrix (**H**). So, for the additive and dominant effects, **g**_**A**_ **=** **H**_**A**_**a** and **g**_**D**_ **=** **H**_**D**_**d: :**

$$H_{A}=\left(\begin{array}{c} h_{\text{A1}}\\ \ldots \\ h_{\text{Ak}}\; \end{array}\right)\text{ and }H_{D}=\left(\begin{array}{c} h_{\text{D1}}\\ \ldots \\ h_{\text{Dk}}\; \end{array}\right)$$

Where each **h** vector is composed by *n* (number of SNP markers) elements (**h**_Ai_ = {*h*_*Ai*1_, *h*_*Ai*2_, …, *h*_*Ain*_} and **h**_Di_ = {*h*_*Di*1_, *h*_*Di*2_, …, *h*_*Din*_}) and **a** and **d** are the vectors of the SNP additive and dominant effects. These **h**_Ai_ and **h**_Di_vectors can be defined in several ways, depending of the reference point or the assumption of the Hardy-Weinberg equilibrium, among others. However, orthogonal partitioning of variances must follow the NOIA approach (Alvarez-Castro and Carlborg, 2007):

$$\begin{array}{l} h_{Aij}=\{\begin{array}{c} -\left(-p_{12j}-2p_{22j}\right)\;A_{1}A_{1}\\ -\left(1-p_{12j}-2p_{22j}\right)\;A_{1}A_{2}\\ -\left(2-p_{12j}-2p_{22j}\right)\;A_{2}A_{2} \end{array}\\ h_{Dij}=\{\begin{array}{c} -\frac{2p_{12j}p_{22j}}{p_{11j}\;+\;p_{22j}-{\left(p_{11j}-p_{22j}\right)}^{2}}\;A_{1}A_{1}\\ \frac{4p_{11j}p_{22j}}{p_{11j}\;+\;p_{22j}-{\left(p_{11j}-p_{22j}\right)}^{2}}\;A_{1}A_{2}\\ -\frac{2p_{11j}p_{12j}}{p_{11j}\;+\;p_{22j}-{\left(p_{11j}-p_{22j}\right)}^{2}}\;A_{2}A_{2} \end{array} \end{array}$$

Therefore, and under the assumption that SNP additive or dominant effects follow a Gaussian distribution, the additive and dominant “genomic” (co) variance relationship matrices can be computed as:

$$Cov\left(g_{A}\right)=\;\frac{H_{A}H_{A}^{\prime }}{tr\;\left(H_{A}H_{A}^{\prime }\right)/n}\sigma_{A}^{2}\;Cov\left(g_{D}\right)\;=\;\frac{H_{D}H_{D}^{\prime }}{tr\;\left(H_{D}H_{D}^{\prime }\right)/n}\sigma_{D}^{2}$$

where the division by traces standardizes the variance components to an ideal infinite “unrelated” population. For second order epistatic effects (**g**_AA_, **g**_AD_, and **g**_DD_), Alvarez-Castro and Carlborg (2007) proved that:

$$h_{\text{AAij}}=h_{\text{Ai}}⊗h_{\text{Aj}}\;h_{\text{ADij}}=h_{\text{Ai}}⊗h_{\text{Dj}}\;h_{\text{DDij}}=h_{\text{Di}}⊗h_{\text{Dj}}$$

and, as a consequence, the matrices **H**_AA_, **H**_AD_ and **H**_DD_ can be written as:

$$\begin{array}{l} H_{AA}=\left(\begin{array}{c} h_{A1}⊗h_{A1}\\ h_{A2}⊗h_{A2}\\ .\\ h_{An}⊗h_{An} \end{array}\right)H_{AD}=\left(\begin{array}{c} h_{A1}⊗h_{D1}\\ h_{A2}⊗h_{D2}\\ .\\ h_{An}⊗h_{Dn} \end{array}\right)\\ H_{DD}=\left(\begin{array}{c} h_{D1}⊗h_{D1}\\ h_{D2}⊗h_{D2}\\ .\\ h_{Dn}⊗h_{Dn} \end{array}\right) \end{array}$$

and, as before, under the assumption of Gaussian distribution of second-order epistatic effects, the covariance between them can be calculated as:

$$\begin{array}{l} Cov\left(g_{AA}\right)=\;\frac{H_{AA}H_{AA}^{\prime }}{tr\left(H_{AA}H_{AA}^{\prime }\right)/n}\sigma_{AA}^{2}\;=\;G_{AA}\sigma_{AA}^{2}\\ Cov\left(g_{AD}\right)=\;\frac{H_{AD}H_{AD}^{\prime }}{tr\left(H_{AD}H_{AD}^{\prime }\right)/n}\sigma_{AD}^{2}\;=\;G_{AD}\sigma_{AD}^{2}\\ Cov\left(g_{DD}\right)=\;\frac{H_{DD}H_{DD}^{\prime }}{tr\left(H_{DD}H_{DD}^{\prime }\right)/n}\sigma_{DD}^{2}\;=\;G_{DD}\sigma_{DD}^{2} \end{array}$$

and the covariance between any higher order epistatic effects must be:

$$Cov\left(g_{ijk}\right)=\;\frac{H_{ijk}H_{ijk}^{\prime }}{tr\left(H_{ijk}H_{ijk}^{\prime }\right)/n}\sigma_{ijk}^{2}\;=\;G_{ijk}\sigma_{ijk}^{2}$$

However, **H** matrices are extremely large and calculation of **HH′** cross-products is computationally expensive; each **H** matrix has as many columns as marker interactions and as many rows as individuals. Nevertheless, Vitezica et al. (2017) provided an algebraic shortcut that allows calculation from the additive and dominance matrices, described above, as:

$$\begin{array}{l} Cov\left(g_{AA}\right)=\frac{G_{A}\;\circ \;G_{A}}{tr\left(G_{A}\;\circ \;G_{A}\right)/n\;}\sigma_{AA}^{2}=G_{AA}\sigma_{AA}^{2}\\ Cov\left(g_{AD}\right)=\frac{G_{A}\;\circ \;G_{D}}{tr\left(G_{A}\;\circ \;G_{D}\right)/n\;}\sigma_{AD}^{2}=G_{AD}\sigma_{AD}^{2}\\ Cov\left(g_{DD}\right)=\frac{G_{D}\;\circ \;G_{D}}{tr\left(G_{D}\;\circ \;G_{D}\right)/n\;}\sigma_{DD}^{2}=G_{DD}\sigma_{DD}^{2} \end{array}$$

For higher order interactions the results are equivalent. As an example, the covariance matrix for the AAD epistatic interaction can be calculated as:

$$Cov\left(g_{AAD}\right)=\frac{G_{A}\;\circ \;G{\;}_{D}\;\circ \;G_{D}}{tr\left(G_{A}\;\circ \;G_{D}\;\circ \;G_{D}\right)/n\;}\sigma_{ADD}^{2}=G_{ADD}\sigma_{ADD}^{2}$$

It should be noted that **G** **∘** **G**… products tend to **I** and higher order epistatic effects tend to be confused with residuals. Nevertheless, this orthogonal approach assumes linkage equilibrium between SNP molecular markers. Linkage disequilibrium (LD) modifies the distribution of the variance into additive, dominance and epistatic components, and orthogonal partition is not possible (Hill and Maki-Tanila, 2015). In outbred populations, substantial LD is present only between polymorphisms in tight linkage (Hill and Maki-Tanila, 2015). However, whilst the distribution of epistatic effects is still unclear (Wei et al., 2015, there is evidence of epistatic interactions between linked loci (Lynch, 1991). Alternative approaches, such as those of Akdemir and Jannick (2015) and Akdemir et al. (2017) have been developed to define locally epistatic relationship matrices. These studies used a RKHS (Reproducing Kernel Hilbert Space) to define these matrices and average them.

The RKHS approach to model epistatic interactions relies on the idea that the relationship between phenotypes and genotypes may not be linear (Gianola et al., 2006; de los Campos et al., 2009a). The main objective is to predict the performance of each individual given its marker genotype through a function that maps the genotypes into phenotypic responses. One of the simplest methods is to consider that this function is linear and, consequently, the results are equivalent to the GBLUP approach. Nevertheless, the power of the Kernel concept relies on the possibility of using alternative functions of marker genotypes. In short, RKHS procedures result in some non-parametric functions g() of a SNP markers set (**X**):

y=μ+g(X)+e

and define a cost function to minimize

$$J={\left(y-g\left(X\right)\right)}^{\prime }\left(y-g\left(X\right)\right)+\lambda {\left\|g\left(X\right)\right\|}_{H}^{2}$$

where the term $||g\left(\text{X}\right)|{|}_{H}^{2}$ is a norm under a Hilbert space. Kimeldorf and Wahba (1971) found that **g**(**X**) can be reformulated as:

$$g\left(X\right)=\alpha_{0}+\sum_{i\;=\;1}^{n}\alpha_{i}K\left(x-x_{i}\right)$$

where **K** is a positive semi-definite matrix that meets the requisites of a Kernel Matrix. It defines the similarity between individuals and meets the distance requirements in a Hilbert space (Wootters, 1981). The performance of the method depends on an adequate choice of **K** that can be chosen from among a very large number of options. The easiest RKHS option is to use the genealogical (**A**) or genomic (**G**) relationship matrices as kernel matrices (Rodríguez-Ramilo et al., 2014), this leads to the standard BLUP or the GBLUP as particular cases of RKHS. However, they only are able to capture the additive genetic variation and if the model tries to accommodate dominance or epistatic interactions, an alternative Kernel matrix has to be implemented for a pair of SNP vectors of two individuals (**x** and **x**′). Most kernels proposed so far (Gianola et al., 2006; Piepho, 2009; Morota et al., 2013; Tusell et al., 2014) consider the similarity across individuals within loci (i.e., similarities within loci are summed). Using Taylor series expansions, it can be shown that kernels of this type are a weighted sum of the additive (**G**) and dominance covariance matrices (**D**), and therefore implicitly account for dominance (Piepho, 2009). However, these kernels do not consider joint similarity across loci. A kernel that includes epistasis should measure similarities simultaneously between pairs, triplets etc., of loci across individuals, as described in Jiang and Reif (2015) and Martini et al. (2016).

## Applications of genomic selection with non-additive genetic effects

### Predictive performance

The most direct application of the genomic prediction models is to predict the performance of an individual for continuous or categorical phenotypes. Here the introduction of non-additive genetic effects in the procedures of prediction becomes relevant, as the main objective is to predict performance conditioned on the genotype of the individual, despite the additive, dominant or epistatic gene action. In fact, simulation studies show up to 17% more accurate predictions based on the sum of additive and dominance effects compared to prediction based on only additive effects (Wellmann and Bennewitz, 2012; Da et al., 2014). However, the performance of semi-parametric or non-parametric approaches such as RKHS methods seems to be appropriate because they are designed to maximize predicting ability over a given individual and not to predict the future performance of the progeny; they are also designed to capture complex and non-explicit interactions. Moreover, some new research fields have merged with genomic evaluation for predicting future performance, examples include: microbiomics (Ramayo-Caldas et al., 2016; Yang et al., 2017), metabolomics (Fontanesi, 2016) and precision farming (Banhazi et al., 2012). Over time they will provide a global picture of the genetic and environmental circumstances that affect the future performance of individuals and they will contribute to the development of more accurate prediction models.

### Mate allocation

In the past, there was a strong belief in “nicking”: pairs of individuals that, wisely selected, would give rise to very efficient offspring (Lush, 1943). In terms of quantitative genetics, the existence of “nicking” would imply that there is large variance of dominant deviations (or epistasis) compared to the variance of breeding values, something that finally turned out to be generally false. Even so, there is room for mate allocation within a population (Toro and Varona, 2010). Under models that include dominance effects, the output of the genomic selection procedure can be used to calculate the prediction of performance of future mating (G_ij_) between the *ith* and *jth* individual as:

$$\begin{array}{l} E\left(G_{ij}\right)=\sum_{k\;=\;1}^{n}[pr_{ijk}\left(A_{1}A_{1}\right){\hat{a}}_{J}+pr_{ijk}\left(A_{1}A_{2}\right){\hat{d}}_{J}\\ \;-pr_{ijk}\left(A_{2}A_{2}\right){\hat{a}}_{J}] \end{array}$$

where *pr*_*ijk*_(*A*_*1*_*A*_*1*_), *pr*_*ijk*_(*A*_*1*_*A*_*2*_), and *pr*_*ijk*_(*A*_*2*_*A*_*2*_) are the probabilities of the genotypes *A*_*1*_*A*_*1*_, *A*_*1*_*A*_*2*_, and A_2_A_2_ for the combination of the *ith* and *jth* individual and the *kth* marker, â_*k*_ and ${\hat{d}}_{k}$ are the estimates of the additive and dominance effects for the same marker and *n* is the number of markers. Later, optimisation procedures like linear programming (Jansen and Wilton, 1985) or heuristic approximations (simulated annealing, Kirkpatrick et al., 1983) can be used to define a set of mates that maximize performance in the future generation. In a simulated example, Toro and Varona (2010) compared random mating vs. mate selection with a model including dominance and found advantages that ranged between 6 and 22% of the expected response. Sun et al. (2013), Ertl et al. (2014), and Aliloo et al. (2017) have confirmed these improvements with dairy cattle data. However, its implementation in livestock populations is limited because it must be taken into account that the accuracy of the prediction of a potential mate will be low and the advantage will be only relevant when traits have a large amount of non-additive genetic variance. In addition, it requires the genotyping of male and females in the population that is not always available. Moreover, the use of models that include more complex interactions, such as models with epistatic effects or non-parametric approaches, is not so immediate. In fact, the predicted performance of a mate should be calculated after integrating the predictive performance over all possible future genotypic configurations of the expected progeny. For epistasis (but not for dominance) these genotypic configurations also depend on recombination fractions across the genome.

### Selection for crossbreeding

There is consensus that profit from non-additive genetic effects in a selection program can be obtained when commercial animals are the product of mating with those that do not participate in the maintenance of a breeding population. The typical way to proceed is to produce two-way or three-way crosses between populations maintained and selected separately (i.e., in pigs). Selection is carried out within lines to benefit from additivity and, in addition, the value of the cross may increase due to the heterosis. Some of the most popular livestock production systems, including pig, poultry, and rabbit production, involve regular crossbreeding schemes, with the aim of capturing the complementarity between the performance of the purebred populations and heterosis. The breeding goal within pure lines is to select individuals to maximize the response in the crossbred population. The traditional approach for this objective was Reciprocal Recurrent Selection—RRS—(Comstock et al., 1949). RRS postulates the selection of individuals in purebred populations based on the performance of their crossbred progeny. If the source of information is the performance of these crossbred progeny, the main drawback of the practical application of RRS is the increase of generation intervals that reduce overall genetic response. In practical terms, the performance of the pure lines is used, and a high genetic purebred/crossbred correlation is sought in order to warrant correct genetic progress (Wei and van der Werf, 1994), however, this may not be the case because of non-additive effects or genotype x environment (G x E) interactions.

The use of genomic information can provide a very useful tool to improve the ability of prediction of breeding values in purebred populations based on crossbred performance without the need to wait for recording crossbred progeny. Ibánez-Escriche et al. (2009) designed a first approach of the use of GS for crossbred performance under a purely additive model. This study defined a breed specific genomic selection model as:

$$y_{i}=\mu +\sum_{j\;=\;1}^{n}\left(t_{ijk}^{S}\alpha_{jk}^{S}+t_{ijl}^{D}\alpha_{jl}^{D}\right)+e_{i}$$

where $t_{ijk}^{S}$ is the SNP allele at the *jth* locus from breed k and received from the sire of the *ith* individual that can take values 0 or 1, and $\alpha_{jk}^{S}$ is the breed-specific substitution effect for the *jth* locus and the *kth* breed. Similarly, $t_{ijl}^{D}$ and $\alpha_{jl}^{D}$ were defined for the alleles received from the dam of the *lth* breed. The objective of this approach was to estimate allele substitution effects within breed. Even under the assumption of absence of G x E interactions, SNP allele substitution effects may differ between populations due to: (1) Specific population patterns of linkage disequilibrium with the QTL, or (2) The presence of genotypic dominance effects. The allelic substitution effects of the A (or B) population (α_*A*_ or α_*B*_) on performance of A x B depends on the biological additive (a) and dominance (d) effects, and the allelic frequencies of B–p_B_- (or A–p_A_ -) as α_*A*_ = *a* + (1 − 2*p*_*B*_) *d* or α_*B*_ = *a* + (1 − 2*p*_*A*_) *d*). Under dominance, Kinghorn et al. (2010) demonstrated a clear advantage of this approach, assuming the estimation of SNP effects was perfect. This model has been expanded by Sevillano et al. (2017) to a three-way crossbreeding scheme, after the evaluation of a procedure to trace the breed-of-origin of alleles in three-way crossbred animals (Sevillano et al., 2016). This is an example of the “partial genetic” approach (substitution effects defined within populations). Stuber and Cockerham (1966) showed that gene substitution effects can be defined within populations or across populations, and, if all the (non-additive) effects are accounted for, both approaches are equivalent. Christensen et al. (2015) proposed an alternative model called the “common genetic” approach. Both models were compared by Xiang et al. (2016, 2017) in the same data set with very similar results, but more research is still needed.

Crossbreeding implies mating between individuals of parental populations and a formal description of the additive and dominance variance in the crossbred population is required to evaluate the relevance of mate allocation when the crossbreds are generated. Toosi et al. (2010) and Zeng et al. (2013) extended the aforementioned model to include additive and dominance effects and proved (in both cases with simulated data) its superiority over the strictly additive model if dominance variance is present. These results were confirmed by Esfandyari et al. (2015), who proved that the response to selection for crossbreeding performance is increased by training on crossbred genotypes and phenotypes, and by tracking the allele line origin when pure lines are not closely related. Later, Vitezica et al. (2016) described the substitution effects and dominance deviations within the scope of an F1 population and showed that the additive and dominant variance in a crossbred population is:

$$\begin{array}{l} \sigma_{A\left(A\right)}^{2}=2p_{A}q_{A}\alpha_{A}^{2}=2[p_{A}q_{A}a^{2}+2p_{A}q_{A}\left(q_{B}-p_{B}\right)ad\\ \;+p_{A}q_{A}{\left(q_{B}-p_{B}\right)}^{2}d^{2}]\\ \sigma_{A\left(A\right)}^{2}=\;2p_{A}q_{A}{\left[a+\left(q_{B}-p_{B}\right)d\right]}^{2}\\ \sigma_{A\left(B\right)}^{2}=2p_{B}q_{B}\alpha_{B}^{2}=2\;[p_{B}q_{B}a^{2}+2p_{B}q_{B}\left(q_{A}-p_{A}\right)\;ad\\ \;+p_{B}q_{B}{\left(q_{A}-p_{A}\right)}^{2}d^{2}\;]\\ \sigma_{A\left(B\right)}^{2}=2p_{B}q_{B}{\left[a+\left(q_{A}-p_{A}\right)d\right]}^{2}\\ \;\sigma_{D}^{2}=4p_{A}q_{A}p_{B}q_{B}d^{2} \end{array}$$

where $\sigma_{A\left(A\right)}^{2}\text{ and }\sigma_{A\left(B\right)}^{2}$ are the additive variance generated by the purebred populations A and B, respectively, $\sigma_{D}^{2}$ is the dominance variance, *p*_*A*_, *q*_*A*_, *p*_*B*_and *q*_*B*_ are the allelic frequencies in purebred populations, and *a* and *d* are the additive and dominance effects.

However, all these approaches assume that the additive and dominance effects have the same magnitude in pure and crossbred populations and this implies an absence of G x E interaction. To avoid this restriction, Vitezica et al. (2016) and Xiang et al. (2016) proposed a multivariate genomic BLUP that is capable of considering different additive and dominance effects and their correlations between pure and crossbred populations.

### Selection in purebred populations

The response to selection in purebred populations depends on the magnitude of the additive variance and on the prediction of the additive breeding values for the candidates for reproduction. It is usually assumed that it is not worth selecting individuals with the highest dominance values because they will go back to zero as a result of random mating. However, Toro (1993, 1998) proposed two mating strategies that can be used to take advantage of dominance in a closed population. The first (Toro, 1993), was a method that basically consists of performing two types of mating: (a) minimum coancestry mating in order to obtain the progenies that will constitute the commercial population and will also be utilized for testing, and (b) maximum coancestry mating from which the breeding population will be maintained. Toro's second strategy (Toro, 1998) advocates the use of the selection of grandparental combinations. Both strategies are analogous with reciprocal-recurrent selection (Comstock et al., 1949) in that they rely on the crucial distinction between commercial and breeding populations. Nevertheless, they have been exclusively tested by simulation and with a reduced set of genes with known additive and dominance effect. Their efficiency has yet to be verified using a large number of SNP markers.

## Final remarks

Despite huge efforts in the development of statistical models for the implementation of genomic selection with non-additive effects, there are still some issues that have to be dealt with before the use of these models in genomic evaluation becomes standard. A major obstacle is the lack of serious testing as this requires extensive data sets with genotypes and phenotypes, and these data sets are rare. In fact, non-additive genetic variance is expected to be low for most traits (Crow, 2010; Hill et al., 2010), with the exception of fitness related traits. Therefore, the inclusion of non-additive effects in genomic selection models will provide very low (or negligible) improvement in the genetic response or the ability of prediction.

Non-additive effects are easily incorporated into GBLUP procedures (Vitezica et al., 2013, 2017) but efforts must be made to define a single-step approach (Aguilar et al., 2010) that is able to use phenotypic data from non-genotyped individuals and the complete genealogical information of breeding schemes. The major limitation of the GBLUP or single-step approaches is the calculation of the inverse of the genomic relationship matrices (**G**), the introduction of non-additive effects will involve the calculation of the inverse of additional matrices related with dominance or epistatic effects. Nevertheless, this is really a constraint in populations with a large number of genotyped individual (i.e., Holstein), while most of the livestock populations do not suffer for any limitations. In fact, the computational cost for inverting additive and non-additive genomic relationship matrices is equivalent. On the other hand, using current pedigree-based BLUP models based on dominance (de Boer and Hoeschele, 1993) seems futile because the models are computationally complicated.

Recent studies (Xiang et al., 2016) have shown that inbreeding depression can be modeled and included in GS approaches through a covariate with the average individual heterozygosity. Nevertheless, this approach only considers the effects of the dominance in inbreeding depression and the role of epistatic interactions in inbreeding depression (Minvielle, 1987) has not yet been studied. However, directional dominance is not necessary requisite for having a substantial dominance variance. In fact it would be interesting to know if there are traits with substantial dominance variance and without inbreeding depression, because they would be good candidates for successful strategies of using dominance. In addition, it should be mentioned that the genetic architecture of non-additive genetic effects and its relationship with inbreeding depression and heterosis is a relevant subject of future research.

The presence of dominance with inbreeding implies the existence of up to five variance components in pedigree-based analysis (Smith and Maki-Tanila, 1990; de Boer and Hoeschele, 1993): additive; dominance between non-inbred; dominance between inbred; covariance between additive; and, inbred dominance values and inbreeding depression. As far as we know, this model has only been used twice with real data in animal breeding (Shaw and Woolliams, 1999; Fernández et al., 2017); their equivalence with the variance components captured by SNP marker effects has to be clarified.

Finally, the parametric approach for the estimation of epistatic effects (Vitezica et al., 2017) fails when linkage disequilibrium is present. A full description of the effect of the genes and their interactions in populations under linkage disequilibrium and the definition of predictive effects has not been reformulated within the scope of genomic selection. It is unclear what we mean by genetic variances when there is linkage disequilibrium, particularly because linkage disequilibrium is population specific and unstable across generations or subpopulations. Nevertheless, Mäki-Tanila and Hill (2014) showed that when the number of loci increases, epistatic variance disappears. At the same time, the proportion of dominance variance stays the same. Thus, dominance variance is the main non-additive component even with linkage disequilibrium (Hill and Maki-Tanila, 2015).

## Author contributions

LV prepare the initial draft of the review and it was corrected and improved by AL, MT, and ZV. The final manuscript was read and approved by all the authors.

### Conflict of interest statement

The authors declare that the research was conducted in the absence of any commercial or financial relationships that could be construed as a potential conflict of interest.
